# Diagnostic Accuracy of Multiple D-Dimer Cutoff Thresholds and Other Clinically Applicable Biomarkers for the Detection and Radiographic Evaluation of Pulmonary Embolism

**DOI:** 10.3390/arm90040039

**Published:** 2022-08-05

**Authors:** Serafeim Chrysikos, Ourania Papaioannou, Theodoros Karampitsakos, Kyriaki Tavernaraki, Ioanna Thanou, Petros Filippousis, Maria Anyfanti, Georgios Hillas, Argyrios Tzouvelekis, Loukas Thanos, Katerina Dimakou

**Affiliations:** 1 5th Respiratory Medicine Department, “Sotiria” Chest Diseases Hospital, 11527 Athens, Greece; 2 Department of Respiratory Medicine, University Hospital of Patras, 26504 Patras, Greece; 3 Department of Medical Imaging and Interventional Radiology, “Sotiria” Chest Diseases Hospital, 11527 Athens, Greece; 4 ICU, G Gennimatas, General Hospital, 11527 Athens, Greece

**Keywords:** pulmonary embolism, D-dimer, age-adjusted cutoff, computed tomography pulmonary angiography, red cell distribution width

## Abstract

**Highlights:**

- According to recent guidelines for the diagnosis and management of pulmonary embolism (PE), the conventional Dd cutoff (0.5 mg/L) has excellent sensitivity for the diagnosis of PE, although there is a marked decrease in specificity.
- Age-adjusted Dd cutoff thresholds for ruling out diagnoses of PE in emergency departments have significantly improved the performance of Dd testing, especially for the elderly, although little is known about the efficacy to reduce the number of CTPAs that could have been performed.
- The most interesting finding in our study was that the novel proposed Dd cutoff (0.74 mg/L) emerged with increased specificity and equal sensitivity compared with 0.5 mg/L and could have reduced the number of CTPAs that were performed in our institution by 17.2%.
- Ancillary CTPA insights of consolidation/atelectasis and unilateral pleural effusion were significantly associated with PE.

**Abstract:**

Background: Diagnostic work-up of pulmonary embolism (PE) remains a challenge. Methods: We retrospectively studied all patients referred for computed tomography pulmonary angiography (CTPA) with suspicion of PE during a 12-month period (2018). The diagnostic accuracy of different D-dimer (Dd) cutoff thresholds for ruling out PE was evaluated. Furthermore, the association of Dd and red cell distribution width (RDW) with embolus location, CTPA findings, and patient outcome was recorded. Results: One thousand seventeen (*n* = 1017) patients were finally analyzed (mean age: 64.6 years (SD = 11.8), males: 549 (54%)). PE incidence was 18.7%. Central and bilateral embolism was present in 44.7% and 59.5%, respectively. Sensitivity and specificity for conventional and age-adjusted Dd cutoff was 98.2%, 7.9%, and 98.2%, 13.1%, respectively. A cutoff threshold (2.1 mg/L) with the best (64.4%) specificity was identified based on Receiver Operating Characteristics analysis. Moreover, a novel proposed Dd cutoff (0.74 mg/L) emerged with increased specificity (20.5%) and equal sensitivity (97%) compared to 0.5 mg/L, characterized by concurrent reduction (17.2%) in the number of performed CTPAs. Consolidation/atelectasis and unilateral pleural effusion were significantly associated with PE (*p* < 0.05, respectively). Patients with consolidation/atelectasis or intrapulmonary nodule(s)/mass on CTPA exhibited significantly greater median Dd values compared to patients without the aforementioned findings (2.34, (IQR 1.29–4.22) vs. 1.59, (IQR 0.81–2.96), and 2.39, (IQR 1.45–4.45) vs. 1.66, (IQR 0.84–3.12), *p* < 0.001, respectively). RDW was significantly greater in patients who died during hospitalization (*p* = 0.012). Conclusions: Age-adjusted Dd increased diagnostic accuracy of Dd testing without significantly decreasing the need for imaging. The proposed Dd value (0.74 mg/L) showed promise towards reducing considerably the need of CTPA. Multiple radiographic findings have been associated with increased Dd values in our study.

## 1. Introduction

Pulmonary embolism (PE) is a common and potentially lethal condition that usually arises from thrombi originating in the deep venous system of the lower extremities. PE represents the third most common cause of death in hospitalized patients following myocardial infarction and stroke, with the annual incidence rate ranging from 39 to 115 per 100,000 people [1].

Diagnostic work-up of PE in the emergency department remains a challenge, including a series of diagnostic tests, more specifically a combination of clinical decision rules (CDR) and plasma D-dimer (Dd) measurement with Computed Tomography Pulmonary Angiography (CTPA), compression ultrasound (CUS), and ventilation/perfusion (V/Q) lung scan [2,3]. CDR such as Wells and revised Geneva scores, which combined symptoms and physical examination findings with predisposing factors, represent the most frequently applied pretest probability assessment [2,3,4]. However, the sensitivity of both rules (64–79% and 55–74%, respectively) limits their utility in ruling out PE [5,6]. Plasma Dd is a fibrin degradation product due to the simultaneous activation of coagulation and fibrinolysis. Dd test represents an essential tool in diagnosing PE, showing remarkably high sensitivity of ≥95% and a negative predictive value (NPV) of 99% [2,7,8]. Current guidelines suggest that low or intermediate pretest probability and Dd levels below the standard cutoff are sufficient to exclude PE [2,3].

On the other hand, the low specificity of this test, which ranges between 35 and 55%, leads most patients with false-positive results to unnecessary CTPAs [7,8]. In the last years, several studies have validated a new strategy of increasing the specificity of the Dd test, including combined clinical probability assessment with age-adjusted Dd cutoff in order to achieve better screening of patients who should undergo CTPA [9,10,11].

CTPA tends to be the standard of care for detecting PE, showing sensitivity and specificity of 83% and 96%, respectively. Furthermore, CTPA may provide an alternative diagnosis as it allows concurrent evaluation of parenchyma and pleural space [12,13,14,15]. Red cell distribution width (RDW) represents a quantitative indicator of erythrocyte size heterogeneity. Several studies have documented that increased RDW was associated with poor clinical outcomes in many cardiovascular and pulmonary diseases [16,17,18]. In patients with PE, high RDW was associated with worse hemodynamic parameters and early mortality [19].

The study aimed to provide a spherical view of Dd and RDW value and contribute to their legitimate and rationale use in clinical practice. Thus, we evaluated the diagnostic accuracy of different Dd cutoff thresholds for ruling out PE. Furthermore, ancillary CTPA findings in patients with or without PE were recorded. Finally, the association of Dd and RDW with embolus location, CTPA findings, and patient outcome was evaluated.

## 2. Material and Methods

We conducted a retrospective observational study in the “Sotiria” Chest Diseases Hospital, Athens, Greece, which serves as a referral center for chest diseases. The following data were extracted from patients with clinically suspected PE who underwent CTPA during a 12-month period (2018): (a) demographic detail (age, gender); (b) date and imaging result; (c) PE location (unilateral/bilateral, central/peripheral, single/multiple emboli); (d) ancillary CTPA findings (normal parenchyma, pleural effusion, consolidation/atelectasis, infiltrate/wedge-shaped opacity/Ground Glass Opacity (GGO), intrapulmonary nodule/mass, pulmonary fibrosis, mosaic attenuation, advanced emphysema (diffuse centrilobular emphysema without bullae or interstitial disease),and advanced bronchiectasis (severe non-cystic fibrosis bronchiectasis)); (e) Dd and RDW analysis prior to CTPA; (f) days of hospitalization/outcome (improvement/death). Inconclusive CTPA examinations due to poor imaging quality were excluded. This retrospective study was approved by the Institutional Review Board of the “Sotiria” Chest Diseases Hospital (IRB protocol approval: 24000/22.09.21).

### 2.1. CTPA Assessment

All CTPA examinations were performed in a 64-slice CT scanner (Philips Ingenuity Core 64). Following our standard CTPA protocol, all patients received 80–100 mL of iodinated intravenous contrast agent (350 mg/mL). Two senior chest radiologists (K.T., P.F.) evaluated separately all CTPA images. The presence and location (unilateral/bilateral, central/peripheral) of any intraluminal filling defect(s) within the pulmonary arterial tree down to a subsegmental level was recorded, as central PE was defined as the presence of embolus within the main trunk, right/left main, and/or lobar pulmonary arteries. Peripheral PE was defined as the presence of embolus within segmental and subsegmental arteries. Furthermore, parenchymal, and pleural CTPA findings in patients with or without PE were recorded.

### 2.2. D-Dimer and RDW Assays

Plasma Dd was measured by quantitative latex photometric immunoassay (STA Compact Hemostasis System, Stago, Paris, France) using a cutoff of 0.5 mg/L according to the manufacturer. Age-adjusted Dd cutoff was defined as age × 0.01 mg/L in patients 50 years or older. RDW was measured using a Beckman Coulter Automated CBC analyzer. The standard reference range for RDW in our laboratory is between 11.6 and 14.8.

### 2.3. Statistical Analysis

For comparison of proportions, chi-square and Fisher’s exact tests were used. Student *t*-tests were computed for comparison of mean values when the distribution was approximately symmetric and Mann–Whitney test, when distribution was not approximately symmetric. Spearman correlation coefficient explored the association of two continuous variables. Sensitivity (SE), specificity (SP), Negative (NPV) and Positive Predictive values (PPV) were calculated for pre-existed Dd cutoff for the prediction of PE. ROC (Receiver Operating Characteristic) analysis was used to evaluate the predictive ability of Dd for PE and determine new optimal cutoffs. The overall performance of the ROC analysis was quantified by computing area under the curve (AUC). All *p* values reported are two-tailed. Statistical significance was set at 0.05, and analyses were conducted using STATA software (version 11.0, Stata Corp, College Station, California, CA, USA).

## 3. Results

### 3.1. Patient Baseline Characteristics

**Figure 1:**
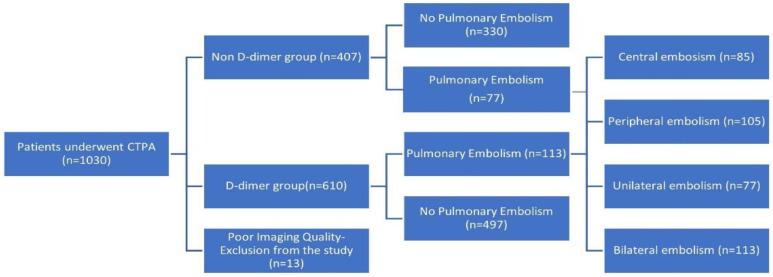
Flowchart of patients enrolled in the study.

Overall, 1030 patients with a suspicion of PE underwent CTPA and were initially registered in the study. Thirteen patients were excluded from the study due to low image quality. A flowchart of patients enrolled in the study is demonstrated in Figure 1. Baseline characteristics and clinical information of patients enrolled in the study are summarized in Table 1. The study population’s mean age was 64.6 years (SD = 11.8), and most patients were males (*n* = 549, 54%). One hundred ninety patients (*n* = 190) were diagnosed with PE, with an overall incidence of 18.7%. Four hundred seven patients (*n* = 407) underwent CTPA without simultaneous Dd analysis, and 77 (18.9%) were diagnosed with PE. Forty-two patients were further evaluated with CTPA, while Dd was below 0.5 mg/L, and two of them (4.8%) were diagnosed with PE.

Significantly greater median Dd values were found in cases with PE compared to cases without PE (3.16, (IQR 1.85–7.82) vs. 1.53, (IQR 0.83–2.87), *p* < 0.001). The median RDW in PE cases was 13.7 (IQR 12.5–15.2). Mean hospitalization concerning patients with PE was 13.2 (SD = 9.0) days, while 8.4% of patients (16/190) died during hospitalization.

### 3.2. Parenchymal and Pleural CTPA Findings in Patients with or without PE

As shown in Table 1, 17.9% (34/190) of patients with PE and 23.1% (191/827) of patients without PE had no abnormal findings on CTPA. Consolidation/Atelectasis was present in 40% (76/190) of patients with PE and 30.8% (255/827) of patients without PE. Ground Glass Opacity/infiltrate/Wedge-shaped opacity, nodule, or mass were all less common. Pleural effusions were present in 41% (78/190) of patients with PE and 36.1% (299/827) of patients without PE. Pleural effusions were more often unilateral than bilateral, and the frequency in patients with or without PE was 29% and 20.8%, respectively. Consolidation/Atelectasis and unilateral pleural effusion were the only findings significantly associated with PE (*p* < 0.05 for both).

### 3.3. Association of Dd and RDW with PE Location, CTPA Findings and Patient Outcome

Table 2 demonstrates the association of Dd value and RDW with PE location, CTPA findings and patient outcome. Significantly greater median Dd values were found in cases with unilateral (5.50, (IQR 3.04–11.89)) and central PE (5.93, (IQR 3.04–12.27)) compared to patients with bilateral (2.20, (IQR 1.40–3.58)) and peripheral PE (2.10, (IQR1.21–3.42)), *p* < 0.001, respectively. Importantly, patients with consolidation/atelectasis or intrapulmonary nodule(s)/mass on CTPA exhibited significantly greater median Dd values compared to patients without consolidation/atelectasis or intrapulmonary nodule(s)/mass, respectively (2.34, (IQR 1.29–4.22) vs. 1.59, (IQR 0.81–2.96), and 2.39, (IQR 1.45–4.45) vs. 1.66, (IQR 0.84–3.12), *p* < 0.001, respectively). Finally, median RDW were significantly greater only in PE patients who died during hospitalization compared to patients who were discharged (15.20, (IQR 14.04–16.50) vs. 13.48, (IQR 12.41–14.50), (*p* = 0.012)).

### 3.4. Diagnostic Accuracy of Different Dd Cutoffs for Ruling out PE

**Figure 2:**
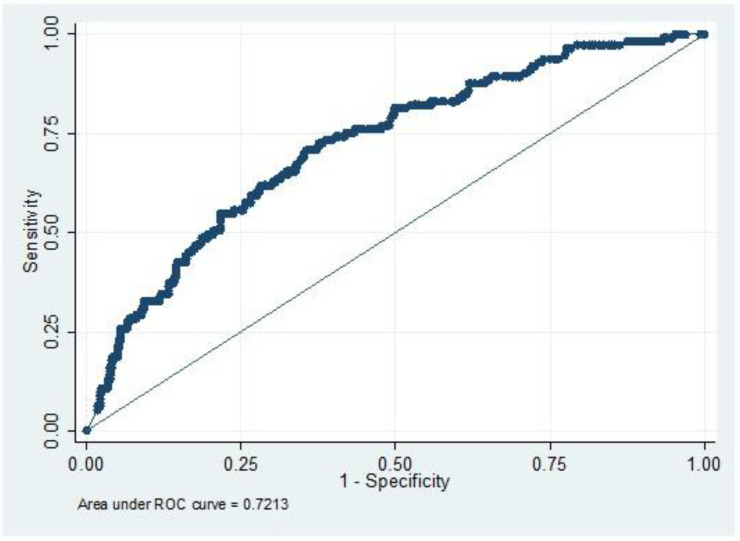
Results from ROC (Receiver Operating Characteristics) analysis for the detection of Pulmonary Embolism from D-dimer testing.

As shown in Table 3, the diagnostic accuracy of Dd using the conventional cutoff of 0.5 mg/L was characterized by 98.2% sensitivity, 7.9% specificity, and 95.2% NPV. When we used age-adjusted cutoff, the sensitivity remained stable (98.2%), while specificity was improved (13.1%). ROC curve was established to confirm a cutoff threshold with the best specificity (Figure 2). Dd levels above 2.1 mg/L have a significant risk for PE incidence (AUC 0.72; 95% CI 0.67–0.77; *p* < 0.001), with a specificity equal to 64.4%. Adopting a new proposed Dd cutoff point of 0.74 mg/L, specificity was increased to 20.5% compared to a conventional cutoff point (0.5 mg/L), leading to a reduction by 17.2% in the number of CTPAs that could have been performed. On the other hand, by implementing the age-adjusted formula, the reduction of the number of CTPAs was 11%.

## 4. Discussion

The findings of our retrospective study, which were conducted in a referral center for chest diseases in Athens, confirm that the age-adjusted Dd cutoff threshold for ruling out diagnosis of PE in the emergency department has significantly improved the performance of Dd testing, especially for the elderly, although little is known about the efficacy to reduce the number of CTPAs that could have been performed. The most interesting finding, in our opinion, was that a novel proposed Dd cutoff (0.74 mg/L) emerged with increased specificity and equal sensitivity compared to 0.5 mg/L and could reduce the number of CTPAs that could have been performed in our institution by 17.2%. Our findings will hopefully contribute towards a more meticulous ordering and interpretation of Dd testing.

According to recent guidelines for diagnosis and management of PE, the conventional Dd cutoff (0.5 mg/L) has excellent sensitivity for the diagnosis of PE, but there is a marked decrease in specificity, so Dd testing is not suggested for confirmation or exclusion of PE [2,3,7]. In addition, it is well documented that the implementation of an age-adjusted Dd cutoff improves specificity without a clinically significant decrease in sensitivity or NPV [2,9,10,11]. It is also well established that the Dd test could be elevated in a range of non-thrombolytic conditions, including pregnancy, malignancies, infections, and the elderly, raising the question of which value of Dd could be the proper one as a marker for further diagnostic algorithm in the case of clinical suspicion of PE [7,8,20].

Our findings confirm previous reports regarding the use of age-adjusted Dd cutoff, highlighting that age-adjusted ones increase specificity without increasing false-negative results [9,21,22,23]. We showed that the absolute percentage of increase in specificity compared with conventional Dd was 5.2%, so the age-adjusted formula is unlikely to significantly decrease the number of patients for whom imaging can be avoided (11% in our study). We also confirm that patients with a higher cutoff (2.1 mg/L) are associated with a significant risk of PE incidence. These findings are consistent with previous studies based on ROC curves illustrating the association between high Dd levels and increased risk for PE [22,24]. In other words, Dd testing may also have a potential prognostic value in PE diagnosis.

CTPA has dramatically changed the diagnostic workup for suspected PE and has become the standard of care for detecting PE. CTPA provides concrete evidence of an embolus location and allows concomitant evaluation of the lung parenchyma and pleural space [14,15]. To our knowledge, our study, compared with previous studies evaluating the pleural and parenchymal findings detected on CTPA in patients with or without PE, enrolls the largest investigating population (*n* = 1017) in which the number of patients with confirmed PE is 190 (18.7%).

Atelectasis with or without consolidation was the most common parenchymal abnormality in the present study, consistent with previous studies [25,26]. Although consolidation is a nonspecific pattern that can result from many different causes, the higher prevalence in patients with PE (*n* = 70, 40%) can be explained by the proposal that it may represent non-Wedge-shaped infarct, oedema or hemorrhage [26].

In contrary to previous studies [25,26,27], we showed that pleural effusions were more often unilateral than bilateral, and the frequency in patients with and without PE was 60.2% and 39.8%, respectively. In our study, among the pleural and parenchymal findings detected on CTPA in patients with or without PE, only consolidation/atelectasis and unilateral pleural effusion showed a statistically significant difference between patients with or without PE. Shah et al., in a study of 92 patients, showed that peripheral Wedge-shaped opacities were the only parenchymal abnormalities significantly associated with PE (25). Ten years later, Karabulut et al., in a study of 128 patients, confirmed that Wedge-shaped opacities and consolidation were significantly associated with PE (26).

In our study, among patients with severe emphysema, pulmonary fibrosis, and advanced bronchiectasis admitted to the hospital with clinical deterioration, PE was detected in 6.3%, 4.7%, and 6.8% of patients, respectively. This reflects the great importance of the involvement of the PE in the differential diagnosis of the acute exacerbation of these entities.

Regarding Dd testing and embolus location, we showed that patients with PE and central/unilateral embolus location presented with increased Dd values. It is well established that accuracy of Dd measurement in patients with suspected PE depends on the embolus present in the largest pulmonary artery [28]. Furthermore, in our study, significantly increased Dd values presented in patients with consolidation/atelectasis and intrapulmonary nodules/mass on CTPA. Our findings are consistent with those of previous reports evaluating factors that could influence Dd levels in cancer patients [21,28]. Finally, in our study, no significant association was found between Dd level and in-hospital mortality. However, a previous observation study showed that elevated Dd levels at diagnosis of PE are associated with an increased risk of death [29,30,31].

With regards to RDW, our study showed a strong correlation between RDW and in-hospital mortality. RDW represents a quantitative indicator of erythrocyte size heterogeneity. Several studies have documented that increased RDW was associated with poor clinical outcomes in many cardiovascular and pulmonary diseases [16,17] including PE [19]. Increased RDW seems to represent a biomarker of early hypoxemia [16,18]. Our results are in line with previous studies [17,19], which documented that increased RDW may potentially serve as an independent predictor of higher mortality in PE patients. In other words, RDW may represent an alternative marker for risk stratification in patients with PE; however, further investigation is needed. Concerning significantly lower RDW in patients with PE and consolidation/atelectasis, it is well established that based on erythrocytes survival (120 days), RDW seem to reflect chronic hypoxia, and thus it is rational that acute diseases such as those leading to consolidation will not increase substantially RDW [16].

Our study exhibited some limitations. First, it presented the inherent weakness of a retrospective study. Data concerning smoking status, further diagnostic evaluation of the study population as well as RDW and outcome of non-PE patients were not available. Secondly, we did not consider the patient’s pretest clinical probability and risk stratification. In our study, 440 patients with suspicion of PE underwent CTPA without a concomitant Dd analysis. This approach is appropriate for patients with high pretest clinical probability or when Dd is expected to be elevated due to comorbidities. Another point that should be considered is the possibility that patients with high clinical probability underwent Dd analysis in contrast to current guidelines. Finally, another limitation of our study is the lack of cross validation for a 0.74 Dd cutoff threshold.

## 5. Conclusions

Our study showed that implementation of an age-adjusted formula for ruling out PE increased diagnostic accuracy of Dd testing without a significant decrease in the number of patients for whom imaging could be avoided. Our proposed Dd value of 0.74 mg/L showed promise towards reducing considerably the need of CTPA but requires further studies before it can be applied clinically. Ancillary CTPA insights of consolidation/atelectasis and unilateral pleural effusion were significantly associated with PE. A strong correlation between plasma Dd and embolus location (central/unilateral), as well as with ancillary findings (consolidation/atelectasis and intrapulmonary nodule(s)/mass), was documented.

Clinical Implications/Future Direction

Taken together, our study by no means indicates that a specific Dd cutoff threshold can guide a diagnostic decision. On the contrary, demonstration of the diagnostic accuracy of multiple Dd cutoff thresholds and presentation of multiple radiographic findings able to lead to increased Dd values offer a more spherical view to clinicians. This report represents another fruitful tool towards meticulous ordering and interpretation of Dd testing. Further studies are needed to evaluate our findings.

## Figures and Tables

**Table 1:** Baseline characteristics and clinical information of patients enrolled in the study.

Characteristics	Presence of PE (*n*,%)	Absence of PE (*n*, %)	*p* Value
Number of patients	190 (18.7)	827 (81.3)	
Age, mean (SD)	64.1 (16.1)	63.6(16.5)	0.703
Gender			
Males	106 (55.8)	443 (52.7)	0.579
Females	84 (44.2)	384 (45.7)	
D-dimer, median (IQR)	3.16 (1.85–7.82)	1.53 (0.83–2.87)	<0.001
RDW median (IQR) ^1^	13.7 (12.5–15.2)		
CTPA Findings			
Normal pleuro-parenchymal findings	34 (17.9)	191 (23.1)	0.119
Consolidation/Atelectasis	76 (40)	255 (30.8)	0.015
Infiltrate/GGO/Wedge-shaped opacity	37 (19.5)	157 (19)	0.877
Nodule/Mass	37 (19.5)	133 (16)	0.259
Pulmonary fibrosis	9 (4.7)	46 (5.6)	0.650
Mosaic attenuation	10 (5.3)	34 (4.1)	0.482
Advanced emphysema	12 (6.3)	69 (8.3)	0.352
Advanced bronchiectasis	13 (6.8)	91 (11)	0.088
Pleural effusion			
Unilateral	55 (29)	172 (20.8)	0.043
Bilateral	23 (12.1)	127 (15.3)	
Hospitalization (days), mean (SD) ^1^	13.2 (9.0)		
Outcome ^1^			
Improvement	142 (74.73)		
Death	16 (8.4)		

Abbreviations: PE = Pulmonary embolism, CTPA = Computed Tomography Pulmonary Angiography, RDW: Red cell Distribution Width, GGO = Ground Glass Opacity, ^1^ = Referred only to patients with PE. Statistically significant *p*-values are shown in bold.

**Table 2:** Association of D-dimer and RDW with PE location, CTPA findings, and patient outcome.

Variates	D-Dimer Median (IQR)	*p* Value	RDW ^1^ Median (IQR)	*p* Value
PE				
Bilateral	2.20 (1.40–3.58)	<0.001	13.56 (12.6–14.60)	0.983
Unilateral	5.50 (3.04–11.89)		13.7 (12.43–15.20)	
PE				
Peripheral	2.10 (1.21–3.42)	<0.001	13.60 (12.41–14.46)	0.730
Central	5.93 (3.04–12.27)		13.58 (12.59–15.40)	
PE				
Single Embolus	2.19 (1.56–3.16)	0.013	13.67 (12.55–14.30)	0.824
Multiple Emboli	3.60 (1.99–8.56)		13.52 (12.43–15.23)	
Normal pleuro-parenchymal findings	1.05 (0.61–2.17)	<0.001	13.75 (12.43–15.20)	0.787
Any parenchymal abnormality	1.97 (1.09–3.53)		13.6 (12.59–14.60)	
Consolidation/Atelectasis				
Present	2.34 (1.29–4.22)	<0.001	12.95 (12.03–14.44)	0.020
Absent	1.59 (0.81–2.96)		13.75 (12.80–15.20)	
Infiltrate/GGO/Wedge-shaped opacity				
Present	1.85 (0.96–3.10)	0.476	13.29 (12.59–14.44)	0.435
Absent	1.74 (0.91–3.32)		13.70 (12.43–15.23)	
Nodule/Mass				
Present	2.39 (1.45–4.45)	<0.001	13.87 (13.48–17.40)	0.118
Absent	1.66 (0.84–3.12)		13.51 (12.41–14.60)	
Pulmonary Fibrosis				
Present	1.22 (0.76–2.96)	0.245	17.20 (15.60–18.80)	0.059
Absent	1.8 (0.93–3.32)		13.60 (12.43- 14.60)	
Mosaic Attenuation				
Present	1.97 (1.31–2.98)	0.585	14.16 (12.65–18.6)	0.249
Absent	1.73 (0.91–3.32)		13.56 (12.43–14.6)	
Advanced Emphysema				
Present	1.18 (0.78–2.37)	0.046	13.60 (12.43–16.40)	0.837
Absent	1.80 (0.94–3.41)		13.62 (12.47–14.85)	
Advanced Bronchiectasis				
Present	1.27 (0.83–2.11)	0.004	14.23 (12.59–15.10)	0.983
Absent	1.85 (0.94–3.49)		13.60 (12.43–14.60)	
Pleural effusion				
Unilateral	2.41 (1.51–4.42)	<0.001	13.00 (12.24–13.70)	0.085
Bilateral	2.56 (1.11–3.60)		14.12 (13.34–16.03)	
Outcome^1^				
Improvement	3.04 (1.78–8.04)	0.474	13.48 (12.41–14.50)	0.012
Death	3.95 (3.16–5.50)		15.50 (14.04–16.50)	

Abbreviations: PE = Pulmonary embolism, CTPA = Computed Tomography Pulmonary Angiography, RDW: Red cell Distribution Width, GGO = Ground Glass Opacity, ^1^ = Referred only to patients with PE. Statistically significant *p*-values are shown in bold.

**Table 3:** Diagnostic accuracy of different D-dimer cutoff thresholds for ruling out PE.

	Conventional Cutoff Point (0.5 mg/L)	Age-Adjusted Cutoff Point	Cutoff Point (2.1 mg/L) with the Best Specificity Based on ROC. AUC (95% CI) 0.72 (0.67–0.77)	Proposed Cutoff Point (0.74 mg/L)
Sensitivity %	98.2	98.2	70.8	97.3
(95% CI)	(93.8–99.8)	(93.8–99.8)	(61.5–79.0)	(92.7–99.1)
Specificity %	7.9	13.1	64.4	20.5
(95% CI)	(5,8–10,8)	(10.2–16.4)	(60–68.6)	(16.4–23.7)
PPV %	19.5	20.4	31.1	21.8
(95% CI)	(16.4–23.1)	(17.1–24.1)	(25.5–37.2)	(17.3–24.6)
NPV %	95.2	97.0	90.7	97.1
(95% CI)	(83.8–99.4)	(89.6–99.6)	(87.1–93)	(89.7–99.6)

Abbreviations: PPV = Positive Predictive Value, NPV = Negative Predictive Value, CI = Confidence Interval, AUC = Area under the curve, ROC (Receiver Operating Characteristics).

## Data Availability

The data presented in this study are available on request from the corresponding author. The data are not publicly available due to ethical reasons.
